# Importance of complex blood flow in the assessment of aortic regurgitation severity using phase contrast magnetic resonance imaging

**DOI:** 10.1007/s10554-021-02341-w

**Published:** 2021-07-17

**Authors:** Frida Truedsson, Christian L. Polte, Sinsia A. Gao, Åse A. Johnsson, Odd Bech-Hanssen, Kerstin M. Lagerstrand

**Affiliations:** 1 Department of Radiation Physics, Institute of Clinical Sciences, Sahlgrenska Academy at University of Gothenburg, 413 45 Gothenburg, Sweden; 2 Department of Medical Physics and Biomedical Engineering, Sahlgrenska University Hospital, 413 45 Gothenburg, Sweden; 3 Institute of Medicine, Sahlgrenska Academy at University of Gothenburg, 413 45 Gothenburg, Sweden; 4 Department of Cardiology, Sahlgrenska University Hospital, 413 45 Gothenburg, Sweden; 5 Department of Clinical Physiology, Sahlgrenska University Hospital, 413 45 Gothenburg, Sweden; 6 Department of Radiology, Institute of Clinical Sciences, Sahlgrenska Academy at University of Gothenburg, 413 45 Gothenburg, Sweden; 7 Department of Radiology, Sahlgrenska University Hospital, 413 45 Gothenburg, Sweden; 8 Sahlgrenska University Hospital, MR-Centre, Bruna stråket 13, 413 45 Gothenburg, Sweden

**Keywords:** Phase contrast magnetic resonance imaging, PC-MRI, Aortic regurgitation, Assessment of aortic regurgitation

## Abstract

This study aimed to investigate if and how complex flow influences the assessment of aortic regurgitation (AR) using phase contrast MRI in patients with chronic AR. Patients with moderate (n = 15) and severe (n = 28) chronic AR were categorized into non-complex flow (NCF) or complex flow (CF) based on the presence of systolic backward flow volume. Phase contrast MRI was performed repeatedly at the level of the sinotubular junction (Ao1) and 1 cm distal to the sinotubular junction (Ao2). All AR patients were assessed to have non-severe AR or severe AR (cut-off values: regurgitation volume (RVol) ≥ 60 ml and regurgitation fraction (RF) ≥ 50%) in both measurement positions. The repeatability was significantly lower, i.e. variation was larger, for patients with CF than for NCF (≥ 12 ± 12% versus ≥ 6 ± 4%, *P* ≤ 0.03). For patients with CF, the repeatability was significantly lower at Ao2 compared to Ao1 (≥ 21 ± 20% versus ≥ 12 ± 12%, *P* ≤ 0.02), as well as the assessment of regurgitation (RVol: 42 ± 34 ml versus 54 ± 42 ml, *P* < 0.001; RF: 30 ± 18% versus 34 ± 16%, *P* = 0.01). This was not the case for patients with NCF. The frequency of patients that changed in AR grade from severe to non-severe when the position of the measurement changed from Ao1 to Ao2 was higher for patients with CF compared to NCF (RVol: 5/26 (19%) versus 1/17 (6%), *P* = 0.2; RF: 4/26 (15%) versus 0/17 (0%), *P* = 0.09). Our study shows that complex flow influences the quantification of chronic AR, which can lead to underestimation of AR severity when using PC-MRI.

## Introduction

Aortic regurgitation (AR) is characterized by the diastolic backward flow of blood from the aorta into the left ventricle. Accurate assessment of chronic AR severity is essential for appropriate clinical decision-making, risk prediction, and timing of surgery [1, 2]. Two-dimensional echocardiography is currently the first-line diagnostic tool and uses a combination of qualitative, semi-quantitative and quantitative parameters for the assessment of AR severity [3, 4]. Cardiovascular magnetic resonance imaging (MRI), currently used as a second-line diagnostic tool, can also provide a comprehensive assessment of AR severity [5, 6]. The assessment can be performed directly using phase contrast MRI (PC-MRI) measurements in an image plane orthogonal to the blood flow in the ascending aorta [7]. For some patients with AR, especially those with a dilatation of the ascending aorta and a bicuspid aortic valve (BAV), the aortic flow profile has been shown to be highly complex, displaying eccentric outflow jets and vortical flow [8, 9]. Accordingly, the blood flow displays not only a linear motion but also regions of swirling and rotating flow pattern. As a result, the systolic blood flow in the ascending aorta includes not only the cardiac output but also backward flow from the swirling blood [10, 11]. Similarly, the diastolic blood flow in such complex flow regions displays not only regurgitant flow but also forward flow from swirling blood.

Even though PC-MRI is considered to be accurate and the standard MRI method for the assessment of AR severity, it has been questioned under certain conditions [12]. For example, it has been shown that the quantification of the aortic regurgitant volume (RVol) and fraction (RF) depends on the position of the image plane, with systematically lower regurgitation values at more distal positions [13–15]. This observation has been attributed to the effect of aortic wall compliance, coronary flow, as well as through plane motion of the aortic root [7, 12–15]. Complex flow may be an additional source of error as the PC method only registers blood flow through an orthogonal image plane that is fixed in space. Accordingly, for swirling flow with both through- and in plane flow components, a part of the blood flow volume may not be registered and the measured flow profile may vary over time. To the best of our knowledge, the influence of complex flow on the assessment of AR severity using PC-MRI has so far not been studied.

Consequently, the aim of this study was to investigate if and how complex flow influences the assessment of AR severity using PC-MRI in patients with chronic AR.

## Material and Methods

### Study design and study cohort

The study cohort has previously been described in another publication, characterizing complex flow patterns in patients with chronic AR [11]. Here, the same PC data is used, but for the purpose of investigating the impact of complex flow on the assessment of AR severity using PC-MRI in patients with chronic AR.

The study comprised 43 patients (24–80 years; 7 females) with moderate (n = 15) and severe (n = 28) chronic AR, determined by 2D echocardiography according to the current ASE guidelines [5]. All participants underwent 2D echocardiography and MRI within 4 h. Exclusion criteria for the patients were the presence of ≥ moderate regurgitation in any other valve, the presence of an intra-cardiac shunt or any other form of cardiac disease, as well as irregular heart rhythm.

### MRI examination

The MRI examination was performed on a 1.5 T whole-body Philips scanner (Achieva, Philips Healthcare, Best, The Netherlands) equipped with a multi-channel phase array cardiac coil.

After standardized patient-specific planning, a series of cine-images was performed, first in the short-axis view covering the whole heart without gap from the atrioventricular ring to the apex, followed by cine-images in the common long-axis projections. All cine-images were acquired in accordance with current guidelines using balanced steady-state free precession sequences (TR/TE = 3.4/1.7 ms and flip angle = 60°) with retrospective electrocardiography (ECG) gating (30 phases per cardiac cycle) and parallel imaging (acc factor = 2) during expiratory breath-hold. Typical in-plane spatial resolution was 1.7 × 1.7 mm^2^ with a slice thickness of 8 mm [16].

**Fig. 1:**
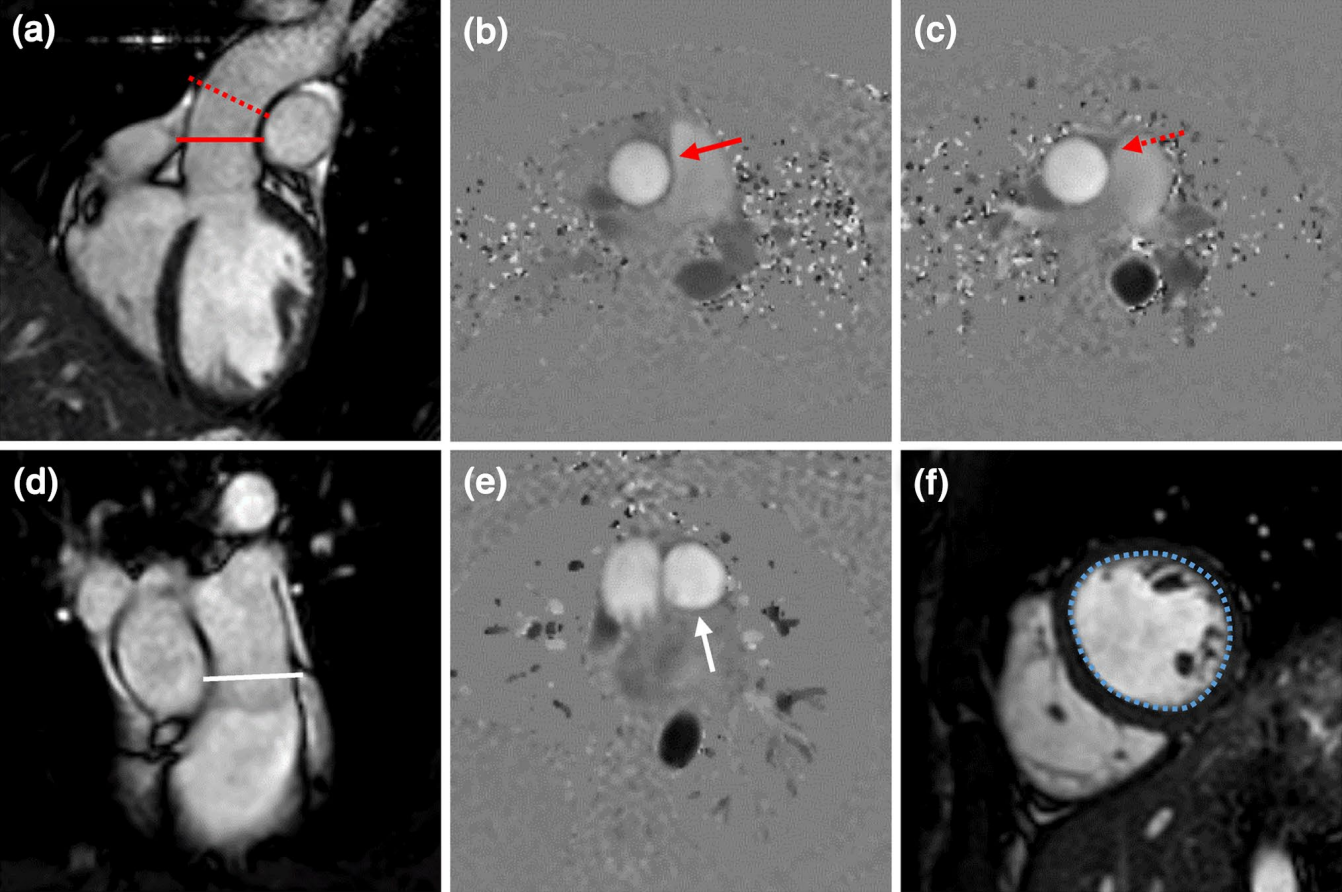
**a** Left-ventricular outflow tract viewed in end-diastole, illustrating the image plane position at the sinotubular (ST)-junction (Ao1: red line) and approximately 1 cm above the ST-junction (Ao2: red dashed line), and corresponding PC images (**b**, **c**) for the quantification of aortic flow (Ao1: red arrow, Ao2: red dashed arrow). **d** Right-ventricular outflow tract viewed in end-diastole, illustrating the image plane position for PC imaging at the pulmonary trunk level (white line), and the corresponding PC image (**e**) for the quantification of pulmonary flow (white arrow). **f** Delineation of the left ventricular endocardial contour (blue dashed lines) in short-axis view to determine left ventricular volumes

ECG-gated PC-MRI measurements during gentle expiratory breath-hold (slice thickness = 8 mm, voxel size = 2.5 × 2.5 mm^2^, TR/TE = 4.8/2.9 ms, BW = 477.8 Hz/pixel, flip angel = 12°, phases per cardiac cycle = 40, acc factor = 2, TFE factor = 4, TFE shots = 13, NSA = 1) were performed in accordance with current guidelines at the level of the sinotubular (ST)-junction (Ao1) as well as 1 cm above the ST-junction (Ao2, Fig. 1) [16]. For indirect quantification of AR, PC-MRI was also performed at the pulmonary trunk just above the pulmonary valve (Fig. 1). The image plane was carefully planned orthogonal to the direction of the blood flow using the flow induced signal void in the functional cine-images as input information. The velocity encoding of the PC-MRI measurement was optimized to the systolic blood flow velocity. Accordingly, velocity encoding was generally set to 150 cm/s for aortic flow and 130 cm/s for pulmonary flow. If the peak blood flow velocity exceeded or was lower than 80% of the chosen velocity encoding level, the measurement was excluded, and a new measurement was performed with adjusted velocity encoding. All PC-MRI measurements with accepted velocity encoding were repeated (M1: first measurement, M2: second measurement) for determination of intra-measurement variability, *i.e.* repeatability.

In all PC-MRI measurements, the phase encoding was chosen in the narrowest anatomic direction, ensuring that no wraparound artifacts affected the interpretation of the images. Special care was taken to improve the temporal resolution by shortening the repetition time and turbo factor of the measurement. The measurements were acquired at the isocenter of the magnet to minimize magnetic field inhomogeneities [12]. Finally, effective compensation for background velocity offsets was performed by the scanner and post-acquisition by means of adaptive image filtering. After compensation, the background offset was below the limit of acceptance (0.6 cm/s) [17].

### Image analysis

Analysis of the MR images was performed using ViewForum (Easy Vision, software release 5.1.7.1, Philips Healthcare, Best, The Netherlands). Left ventricular volumes were obtained by manual tracing of the endocardial contour in the successive slices of the continuous SA stack. Endocardial contours were subsequently propagated through all phases using a semi-automated tracing algorithm, followed by manual adjustment, if necessary (Fig. 1). Compensation for basal through-plane motion was performed according to a previously described technique by Alfakih et al. [18]. Papillary muscles and trabeculae were included in the left ventricular cavity. The left ventricular stroke volume (LVSV) was calculated by subtracting the end-systolic volume from the end-diastolic volume, where end-diastolic volume and end-systolic volume were computed by the slice summation technique. The RVol was measured directly using PC-MRI at both measurement positions, i.e. Ao1 and Ao2, as well as the pulmonary stroke volume (PuSV) at the right-ventricular outflow tract. Aortic and pulmonary flow volumes were determined by delineating the vessels on the magnitude image, copied onto the phase image, and propagating through all phases using a semi-automated tracing algorithm. Manual adjustment was performed if necessary. The direct quantification of RVol and SV was performed by integrating all velocities within the aortic vessel lumen over the diastolic, and systolic phases of the cardiac cycle, respectively. Then, RF was calculated as the diastolic blood volume relative to the systolic blood volume. The pulmonary stroke volume (PuSV) was calculated as the net pulmonary blood volume over the whole cardiac cycle. An indirect quantification of the RVol was performed by subtracting the PuSV from the LVSV (volumetric method) and used as an internal reference. The aortic diameter at position Ao1 and Ao2 was determined using the magnitude image of the 2D PC-MRI measurements at end-systole. The aortic diameter was also determined by using the trailing to leading edge technique in the cine long axis projections. On the basis of the ascending aorta diameter, the aorta was defined as normal (< 40 mm) or dilated (≥ 40 mm) [19]. The pattern of ascending aortic dilatation was categorized in three phenotypes: no-dilatation phenotype, ascending phenotype (dilated ascending aorta with less dilated root), and root phenotype (dilated root with normal or less dilated ascending aorta) [17].

### Blood flow characterization using advanced PC-MRI analysis

**Fig. 2:**
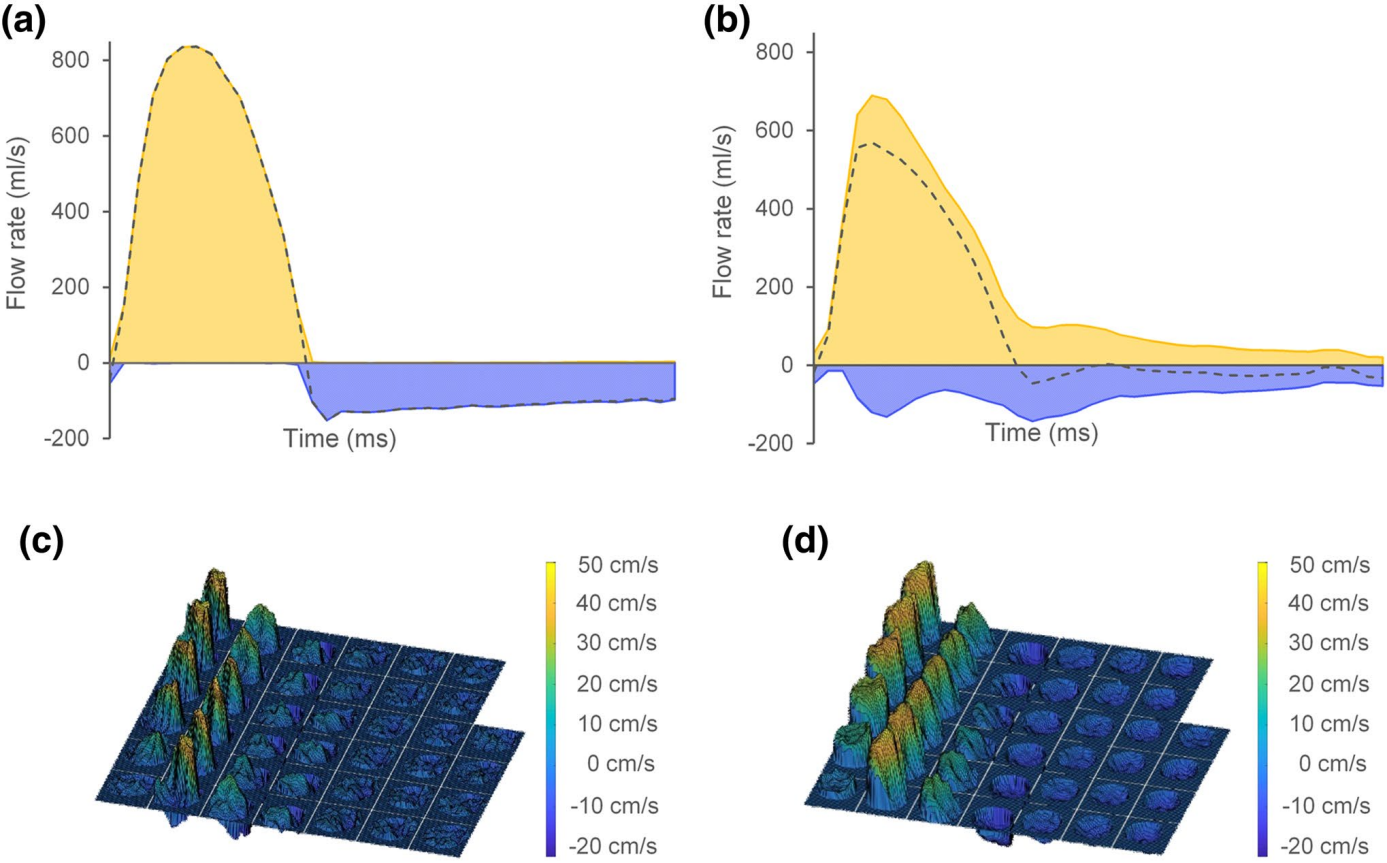
Examples of net (grey dashed lines), forward (orange), and backward (blue) flow rate curves obtained from PC-MRI measurements in AR patients with non-complex (**a**) and complex flow (**b**), including the corresponding velocity profile images (**c**, **d**). The curves showed high forward flow during systole for all subjects. In patients with non-complex flow, regurgitant flow was visible as backward flow only during diastole. For patients with complex flow backward flow was also visible during systole. In the velocity profile images the forward flow is indicated in orange and the backward flow in dark blue. In patients with non-complex flow, the velocity profile images showed homogeneous distribution of the velocities over the vessel area. In patients with complex flow, the distribution of velocities was more heterogenic, displaying eccentric outflow jets during systole and sometimes also during diastole, modifying the distribution of the regurgitant flow jet over the diastolic phases. Also, in some patients with complex flow the velocity profile images showed a helical distribution of velocities over the cardiac phases

Detailed flow analysis was performed in accordance with Barker et al. [10] to characterize and estimate the grade of blood flow complexity in terms of amount of systolic backward flow volume (BFV), associated with swirling flow. The detailed flow analysis was performed using an offline research tool (Segment v1.9 R2046) [20]. First, the net, forward and backward flow rates at the different time frames of the cardiac cycle were calculated. Then, curves describing the net, forward and backward flow rate over the cardiac cycle were plotted. Finally, the BFV during systole (defined from the net flow rate curve as the positive flow interval) was calculated from the backward flow rate curve. Patients were categorized to have either complex flow (CF) or non-complex flow (NCF) based on presence or absence of systolic BFV. In other words, patients with flow rate curves that clearly displayed backward flow beginning earlier than the time point of peak systolic flow were categorized to have CF (Fig. 2) [11]. Patients with no backward flow during systole were categorized to have non-complex flow (NCF).

For comparison, the forward flow volume (FFV) during systole was calculated from the forward flow rate curve. Also, the eccentricity of the outflow jet was estimated as the flow displacement (FD) parameter described by Sigovan et al. [21]. For that purpose, velocity profile images describing the velocity distribution over the vessel area were plotted for all phases of the cardiac cycle (Fig. 2). From these images, FD was calculated as the distance between the center of the lumen and the “center of velocity” as percent of the luminal radius.

Subanalyses were performed to study the effect of the measurement position. For that purpose, the percentage difference of RVol between positions was calculated, and then correlated to systolic BFV, FD and aortic dimension.

### Assessment of AR severity by cardiovascular MRI

All AR patients were assessed to have non-severe AR or severe AR according to current guidelines [2]. Cut-off values for severe AR were: RVol ≥ 60 ml and RF ≥ 50%. The AR assessment was performed for both measurement positions, *i.e.* Ao1 and Ao2, as well as for the repeated measurements, *i.e.* M1 and M2.

### Statistical analysis

Values are expressed as the mean ± standard deviation, unless otherwise stated. The repeatability was calculated as the absolute value of the difference of the two consecutive PC-MRI measurements (|M1 − M2|) as percent of their mean ((M1 + M2)/2). The agreement between the methods was evaluated using the Bland–Altman method, calculating the mean difference (MD) and limits of agreement (LoA = MD ± 1.96 SD) [18]. The degree of linear correlation between the methods, between the systolic BFV and the aortic diameter, between SV and LVSV, as well as between measures in the subanalysis were assessed by the Pearson correlation coefficient (R). The Wilcoxon signed-rank test, at a significant level of *P* < 0.05, was used to test significance between methods and different measurement positions. Mann–Whitney U-test, at a significant level of *P* < 0.05, was used to test difference between different types of flow characteristic (NCF versus CF)). Correction for multiple testing was performed using the Holm step-down method, where the *P* value was adjusted accordingly [22]. Statistical analysis was performed using MATLAB (R2018a, The MathWorks, Inc., Natick, Massachusetts, United States, 2018).

## Results

All subjects were successfully examined with MRI. However, two PC-MRI measurements were not repeated at the distal measurement position Ao2 and were therefore excluded from the repeatability analysis at that position, as well as from the repeatability analysis regarding impact of measurement position.

### Patient characteristic

Patient characteristics are shown in Table 1. A dilated ascending aorta was more frequently present in patients with CF (n = 26) compared to patients with NCF (n = 17). Also, a BAV was more common in patients with CF, and the outflow jets were significantly more eccentric. Furthermore, patients with CF had significantly smaller regurgitation values compared to patients with NCF. No significant differences were found regarding systolic FFV, age, gender and BSA.

**Table 1:** Clinical characteristics of patients with non-complex and complex flow in the ascending aorta [11]

	Non-complex (n = 17)	Complex (n = 26)	*P* value
Clinical and CMR findings			
Age (years)	51 ± 15	50 ± 16	0.8
Male gender (n (%))	15 (88)	21 (81)	0.5
BSA (m^2^)	2.06 ± 0.27	2.0 ± 0.2	0.9
Bicuspid aortic valve (n (%))	5 (29)	17 (65)	0.02
Systolic blood pressure (mmHg)	137 ± 21 (n = 13)	137 ± 23 (n = 13)	0.1
Diastolic blood pressure (mmHg)	58 ± 11 (n = 20)	71 ± 12 (n = 20)	0.007
Heart rate (bpm)	61 ± 10 (n = 16)	63 ± 9	0.6
Ejection fraction (%)	56 ± 6	59 ± 7	0.2
Left ventricular outflow tract (mm)	28 ± 3	30 ± 4	0.03
Sinus Valsalva (mm)	39 ± 5 (n = 16)	42 ± 5	0.1
Sino-tubular junction (mm)	31 ± 5	36 ± 5	0.003
Ascending aorta (mm)	35 ± 6 (n = 16)	44 ± 7	< 0.001
No-dilatation phenotype (no (%))	12 (70)	6 (23)	0.006
Ascending phenotype (no (%))	2 (12)	3 (12)	0.6
Root phenotype (no (%))	3 (18)	19 (73)	< 0.001
Peak aortic velocity (m/s)	16 ± 10	19 ± 11	0.3
Peak Doppler gradient (mmHg)	2 ± 0.5	2 ± 0.6	0.3
Severe AR (n (%))	14 (82)	14 (54)	0.06
PC-MRI data			
Position Ao1			
Ao1 diameter (mm)	33 ± 4	42 ± 6	< 0.001
Ao1 ≥ 40 mm (n (%))	1 (6)	16 (62)	< 0.001
Ao1 ≥ 40 mm + bicuspid aortic valve (n (%))	0 (0)	12 (46)	0.001
RVol_direct_ M1 (ml)	80 ± 38	54 ± 42	0.01
RVol_direct_ M2 (ml)	80 ± 37	54 ± 40	0.01
∆RVol_direct_ [M1-M2] (ml)	-0.01 ± 6	0.5 ± 7	0.8
RF_direct_ M1 (%)	47 ± 14	34 ± 16	0.01
RF_direct_ M2 (%)	47 ± 13	34 ± 17	0.02
∆RF_direct_ [M1-M2] (%)	0.2 ± 4	-0.2 ± 6	0.5
Systolic FFV (ml)	168 ± 43	179 ± 52	0.4
Systolic BFV (ml)	6 ± 4	35 ± 15	< 0.001
Systolic FD	0.15 ± 0.08	0.34 ± 0.09	< 0.001
Position Ao2			
Ao2 diameter (mm)	34 ± 5	45 ± 5	< 0.001
Ao2 ≥ 40 mm (n (%))	3 (18)	21 (81)	< 0.001
Ao2 ≥ 40 mm + bicuspid aortic valve (n (%))	1 (6)	15 (58)	< 0.001
RVol_direct_ M1 (ml)	77 ± 38	42 ± 34	0.004
RVol_direct_ M2 (ml)	77 ± 38	48 ± 37 (n = 24)	0.01
∆RVol_direct_ [M1 − M2] (ml)	-0.04 ± 5	-3 ± 9 (n = 24)	0.5
RF_direct_ M1 (%)	48 ± 16	30 ± 18	0.003
RF_direct_ M2 (%)	48 ± 16	34 ± 19 (n = 24)	0.02
∆RF_direct_ [M1 − M2] (%)	− 0.2 ± 5	− 2 ± 8 (n = 24)	0.8
Systolic FFV (ml)	168 ± 44	160 ± 49	0.5
Systolic BFV (ml)	7 ± 5	30 ± 21	< 0.001
Systolic FD	0.11 ± 0.10	0.36 ± 0.14	< 0.001

Data are presented as mean ± standard deviation (SD). The significance of the differences between patients with non-complex and complex flow in the ascending aorta are presented as *P* values

*Ao1* measurement position at the sinotubular (ST)-junction, *Ao2* measurement position 1 cm above the ST-junction, *BFV* backward flow volume, *BSA* body surface area, *CMR* cardiovascular magnetic resonance, *FD* flow displacement, *FFV* forward flow volume, *M1* first measurement, *M2* second measurement, *RF*_*direct*_ regurgitant fraction obtained using phase contrast magnetic resonance imaging (PC-MRI), *RVol*_*direct*_ regurgitant volume obtained using PC-MRI

**Fig. 3:**
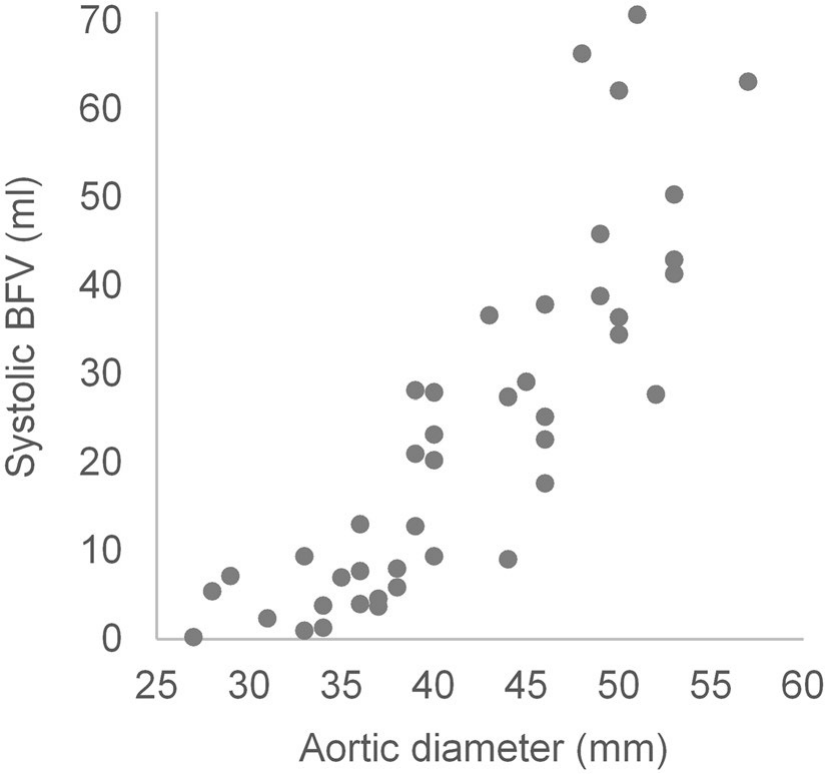
Linear regression between backward flow volume (BFV) during systole and the aortic diameter in patients with chronic aortic regurgitation quantified by the direct method

The grade of flow complexity, as indicated by the systolic BFV, increased with increasing aorta dimension (R = 0.8, *P* < 0.001, Fig. 3). Furthermore, the aorta was significantly larger at the distal measurement position Ao2 (diameter = 41 ± 7 mm) than at the level of the ST-junction Ao1 (diameter = 39 ± 7 mm, *P* < 0.001).

### Internal reference versus direct quantification method

**Fig. 4:**
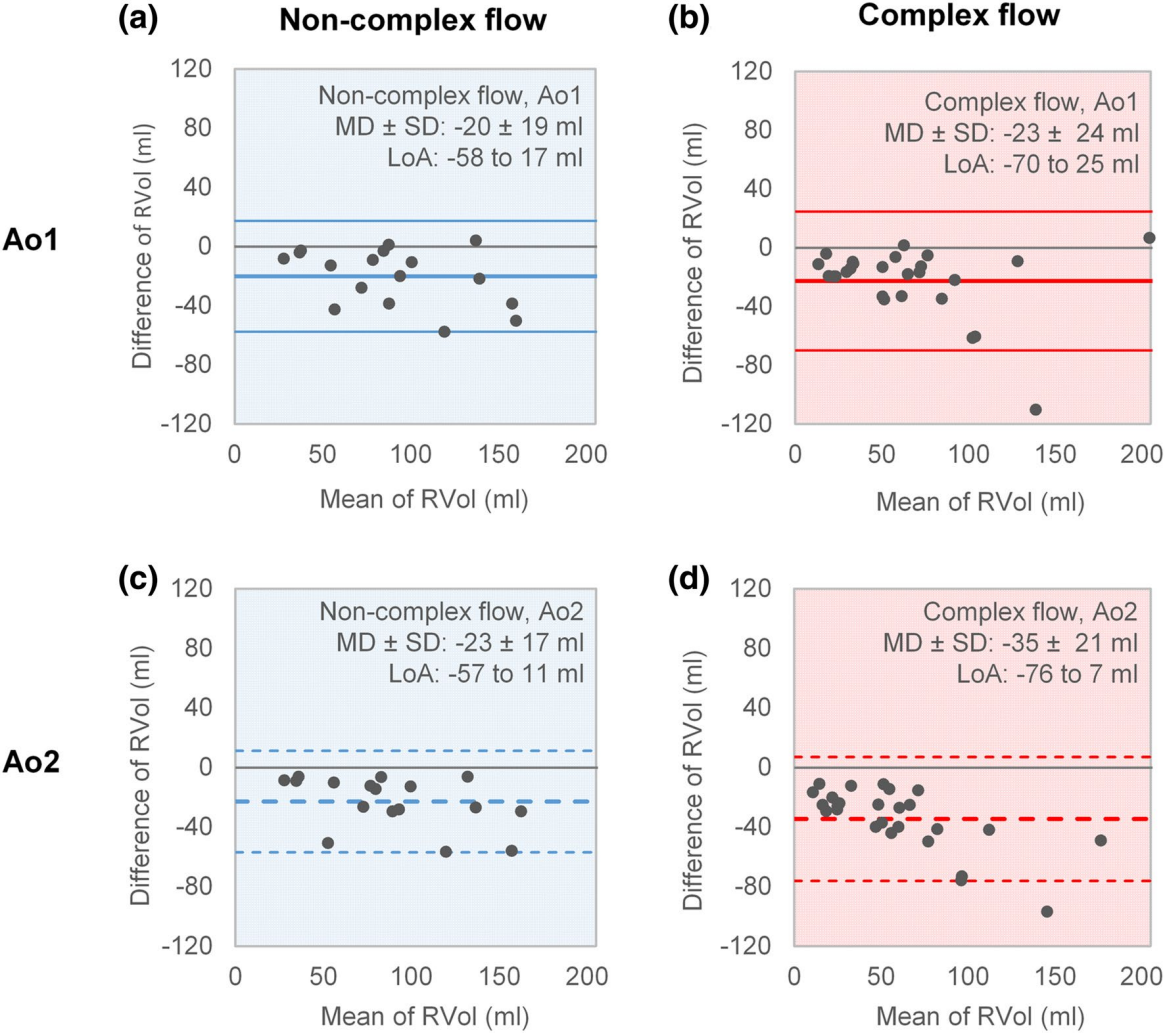
Bland–Altman plots comparing the regurgitant volume obtained by the internal reference method [RVol = left ventricular stroke volume (LVSV) − pulmonary stroke volume (PuSV)] with the direct method (PC-MRI) at measurement position Ao1 (**a**, **b**: top panel; solid lines) and Ao2 (**c**, **d**: bottom panel; dashed lines) for patients with non-complex flow (**a**, **c**: blue panels to the left), and complex flow (**b**, **d**: red panels to the right; NCF: n = 17, CF: n = 26). The colored lines represent the mean relative difference (MD) and 95% limits of agreement (LoA), and the grey line represents zero relative difference. SD, standard deviation

The direct RVol measure correlated with the internal reference for both CF and NCF patients. This was the case for both measurement positions, *i.e.* Ao1 and Ao2 (NCF [Ao1]: R = 0.92, *P* < 0.001; NCF [Ao2]: R = 0.93, *P* < 0.001; CF [Ao1]: R = 0.87, *P* < 0.001; CF [Ao2]: R = 0.93, *P* < 0.001). Overall, the direct method gave significantly smaller RVols than the internal reference method (Table 2). This was most prominent in patients with CF at measurement position Ao2 (Bland–Altman analysis, Fig. 4). At Ao2, the difference between the methods was larger for patients with CF than for patients with NCF (Table 2). Furthermore, the comparison between the SV (forward flow–RVol) determined by PC-MRI and the LVSV calculated by the slice summation technique showed strong correlation (R = 0.95, *P* < 0.001).

**Table 2:** Comparison between the internal reference and the direct quantification method to obtain the regurgitant volume

	Non-complex (n = 17)	Complex (n = 26)	*P* value
RVol_internal reference_ (ml)	100 ± 46	77 ± 49	0.04
Position Ao1			
∆RVol_direct_ (ml)	20 ± 19	23 ± 24	0.7^a^
*P* value (internal reference versus direct)	< 0.001^a^	< 0.001^a^	
∆RVol_direct_ as percent of RVol_internal reference_ (%)	20 ± 15	33 ± 20	0.07^a^
Position Ao2			
∆RVol_direct_ (ml)	23 ± 17	35 ± 21	0.06^a^
*P* value (internal reference versus direct)	< 0.001^a^	< 0.001^a^	
∆RVol_direct_ as percent of RVol_internal reference_ (%)	23 ± 14	51 ± 20	< 0.001^a^

Data are presented as mean ± standard deviation (SD). The significance of the differences between the internal reference and direct method to quantify the RVol, and between patients with non-complex and complex flow in the ascending aorta are presented as *P* values. Otherwise, abbreviations as in Table 1

*RVol*_*internal reference*_ regurgitant volume obtained using an internal reference method [left ventricular stroke volume (LVSV) − pulmonary stroke volume (PuSV)]

^a^Corrected *P* value according to the Holm step-down method [22]

### Repeatability

**Fig. 5:**
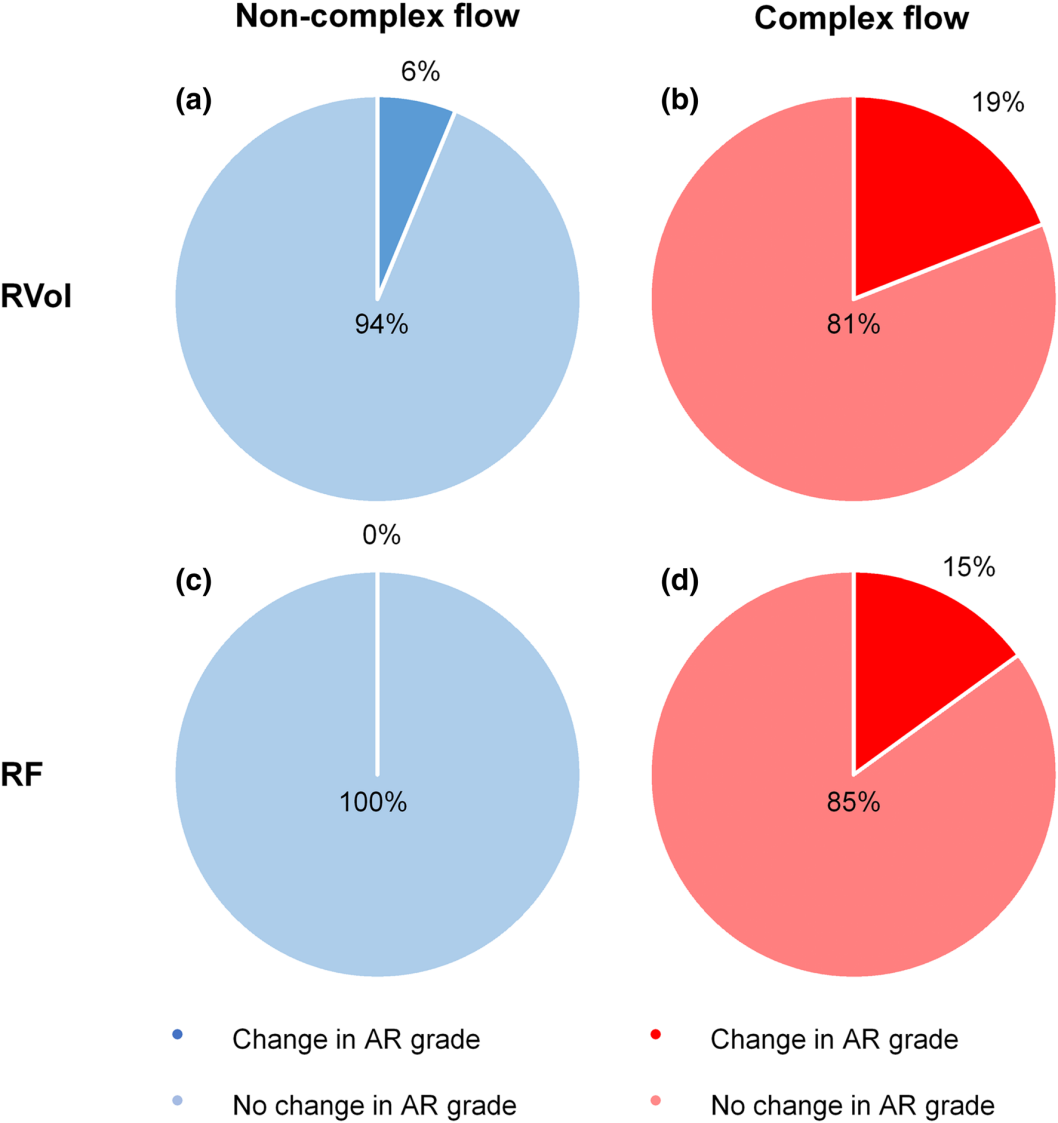
This figure illustrate the frequency of patients that changed in AR grade from non-severe to severe, and vice versa, based on the RVol and RF. Each pie chart represent the comparison between measurement positions (Ao1: sinotubolar-junction, Ao2: 1 cm above the sinotubolar-junction) for patients with non-complex flow (blue) and complex flow (red), separately. Transparent colors represent the frequency of patients that changed in AR grade (from moderate to severe, or reversed), and saturated color represent the frequency of patients that did not change in AR grade. **a** RVol, non-complex flow; **b** RVol, complex flow; **c** RF, non-complex flow; **d** RF, complex flow

The repeatability was significantly lower, i.e. the variability was larger, for patients with CF than for patients with NCF, at both measurement positions (Table 3). However, no systematic difference was found in RVol and RF between measurement 1 and 2 (Tables 1 and 3), and the number of patients that changed in AR grade from non-severe to severe, and vice versa, was not significant (Fig. 5).

**Table 3:** Comparison of repeatability concerning the RVol and RF between patients with non-complex and complex flow in the ascending aorta

	Non-complex (n = 17)	Complex (n = 26)	*P* value
Position Ao1			
Repeatability of RVol_direct_ (%)	6 ± 4	12 ± 12	0.03^a^
Frequency of patients that changed in AR grade (n (%))	1/17 (6)	3/26 (12)	0.6
Repeatability of RF_direct_ (%)	6 ± 6	15 ± 13	0.02^a^
Frequency of patients that changed in AR grade (n (%))	2/17 (12)	4/26 (15)	0.8
Position Ao2			
Repeatability of RVol_direct_ (%)	6 ± 6	21 ± 20 (n = 24)	0.002^a^
Frequency of patients that changed in AR grade (n (%))	1/17 (6)	3/24 (13, n = 24)	0.5
Repeatability of RF_direct_ (%)	8 ± 7	25 ± 22 (n = 24)	0.001^a^
Frequency of patients that changed in AR grade (n (%))	0	2/24 (8, n = 24)	0.2

Data are presented as mean ± standard deviation (SD). The significance of the differences of the repeatability of RVol and RF between patients with non-complex and complex ascending aorta flow are presented as *P* values. Abbreviations as in Table 1

^a^Corrected *P* value according to the Holm step-down method [22]

### Measurement position

No significant influence of measurement position, neither regarding regurgitation values nor regarding repeatability of regurgitation values, was found for patients with NCF (RVol: *P* = 0.3; RF: *P* = 0.7; Repeatability of RVol: *P* = 0.9; Repeatability of RF: *P* = 0.5; the *P* values are corrected for multiple testing [22]; Tables 1 and 3). For patients with CF, however, the regurgitation values were significantly lower and the repeatability of the regurgitation values was significantly reduced at Ao2 in comparison to Ao1 (RVol: *P* < 0.001; RF: *P* = 0.01; Repeatability of RVol: *P* = 0.02; Repeatability of RF: *P* = 0.02; the *P* values are corrected for multiple testing [22]; Tables 1 and 3).

The frequency of patients that changed in AR grade from non-severe to severe, and vice versa, when the position of the measurement changed from Ao1 to Ao2 was higher for patients with CF compared to NCF (RVol: 5/26 (19%) versus 1/17 (6%), *P* = 0.2; RF: 4/26 (15%) versus 0/17 (0%), *P* = 0.09); Fig. 5).

The percentage difference of the RVol between positions and aortic dimension showed only a weak association (R = 0.45, *P* = 0.02). The comparison of the percentage difference of RVol between positions and FD showed a moderate association (R = 0.57, *P* < 0.001). Similar results were found for systolic BFV (R = 0.51, *P* < 0.001).

## Discussion

Our results indicate that complex flow can affect the assessment of AR severity, as indicated by a larger difference in RVol between the direct and indirect reference measurements, as well as in a reduced repeatability. In patients with complex flow, the quantification of the regurgitation values was shown to depend on the aortic dimension and measurement position. Specifically, lower regurgitation values were measured at the most distal measurements position where the aortic dimension was found to be largest.

Most vessels in the body display laminar flow with slight asymmetries close to the branches. However, cardiovascular disease can modify the hemodynamic conditions and change the vascular flow pattern. It has been shown that a BAV is associated with abnormal swirling flow and eccentric outflow jets [9, 23]. Such flow pattern has also been reported in patients with a BAV in combination with a dilated aorta [9]. In this study, patients with complex flow that had a BAV encompassed 65% (17/26) of the cohort, and patients with complex flow that had a BAV in combination with a dilated aorta encompassed 58% (15/26) of the cohort. Accordingly, the majority of the patients with complex flow that had a BAV also had a dilated aorta, and only 8% (2/26) had a BAV without dilatation. Abnormal swirling flow has been reported in patients with an aneurysmatic aorta in the absence of a BAV [24–26]. Present findings are in concordance with these studies, where tricuspid aortic valve patients with complex flow and dilatation of the aorta encompassed 23% (6/26) of the cohort. Thus, the findings suggest that complex flow is not only caused by an aortic valve malformation, but also by the hemodynamics associated with an enlarged aorta [24–26]. That is, abnormal flow may occur when the aorta dimension is substantially enlarged.

Abnormal swirling flow patterns can diminish the accuracy of the regurgitation values, as the technique only measures the flow vector that is perpendicular to the image plane. Accordingly, flow along with the image plane does not contribute to the registered velocity and for oblique flow, thus, there is a risk that part of the volume is not registered which may result in underestimation. Complex flow, with large variation in flow dynamics over the ascending aorta, could also affect the repeatability of the PC-MRI measurement. For such vessels, even a small variation in the measurement position between the repeated measurements could result in a large variation of the regurgitation values. Since the aorta moves with the beating of the heart while the image plane is fixed in space, different portions of the aorta will be enclosed in the image slice at different time points of the cardiac cycle. This will be the case if the heart pulse varies between repeated measurements, which often is the case. This effect was clearly displayed in the present study, where the variation in the regurgitation values was quite high, approximately 10% at ST-junction and 20% 1 cm distal to ST-junction. In relation to the threshold level for severe AR (60 ml) this variation corresponds to 6 ml respectively 12 ml and, thus, should be taken into consideration in the evaluation of AR severity.

The assessment of AR was shown to depend on the position of the measurement. For patients with complex flow, displacement of the measurement plane to a more distal position in the ascending aorta significantly affected the assessment, which may be due to a higher grade of in plane flow at this position, and consequently a higher grade of complex flow. For patients with non-complex flow, no differences were found in the quantification of the regurgitation values between different measurement positions. Others have shown that the accuracy of regurgitation quantification decreases with increased distance to the aortic valve [14, 27]. However, previous work did not stratify between patients with complex and non-complex flow. Thus, the results of the present study suggest that their conclusion only applies on patients with complex flow. In patients with non-complex flow, the image plane can be chosen arbitrary. In clinical practice, this is advantageous as the choice of measuring position sometimes is limited, e.g. close to mechanical heart valves.

In the present study, an internal reference (LVSV–PuSV) was used for comparison with RVol of AR as it is not affected by complex flow in the ascending aorta. The method has a higher variability in comparison to PC-MRI [28], but has lower variability in comparison to other possible methods. For example, the difference between the right ventricular and LVSV offers another reference method that is independent of complex flow in the ascending aorta [29]. However, the variability of right ventricular SV is higher in comparison to PC-MRI PuSV, and as a consequence, the variability of LVSV–right ventricular SV is higher than for LVSV–PuSV [28]. The comparison between the reference and PC-MRI displayed a linear relationship with a systematic offset with lower RVols for the PC-MRI method. Aortic wall compliance as well as through plane motion of the aortic root [7, 12–15] may have contributed to an underestimation of RVol using PC-MRI. The offset may also reflect an overestimation of LVSV, resulting in a larger reference value. This could, for example, be attributed to the inclusion of papillary muscles and trabeculae in the ventricular cavity [30] as well as through-plane motion of the basal slice [31]. Nevertheless, the offset was significantly larger for patients with complex flow, demonstrating the influence of the hemodynamic condition in the ascending aorta on the assessment of AR using PC-MRI.

Phase contrast MRI is an established method that allows non-invasive measurement of blood flow in vessels deep in the body [32–37]. The accuracy of PC-MRI have been thoroughly investigated, and a number of factors that influence the measured phase values have been identified [38]. With compensation for these known problems, the PC-MRI method has been shown to be very accurate [36, 39, 40]. However, we have demonstrated that complex flow can be a source of error and that the flow pattern is associated with dilatation of the ascending aorta. Thus, it’s essential to identify patients with complex flow, i.e. patients with a dilated aorta, as it can potentially lead to underestimation of AR severity, and thereby potentially affect the clinical decision-making and eventually the timing of surgery.

Present findings show that complex flow is strongly associated with dilatation of the aorta. In our study, most patients with aortaic dilatation (≥ 40 mm [19]) presented a complex flow pattern (> 85%). Hence, one should be cautious when AR severity is assessed in patients with aortic dilatation as the resulting flow pattern in the enlarged region can result in reduced diagnostic certainty. As such, aortic dilatation (> 40 mm) could be a simple criterium in the daily clinical routine. Others have developed strategies to improve the diagnostic certainty for AR [41, 42], suggesting that the assessment should rely on multiple MRI parameters- and MRI-specific thresholds. Another recommendation to increase the diagnostic certainty in patients with complex flow is to perform the PC-MRI measurement at a position with less blood flow complexity, preferably at the aortic valve plane level, avoiding the turbulent and accelerated flow regions secondary to the valve leaflets [43]. However, there are other difficulties associated with this method and a better alternative may be to assess the AR severity using the indirect quantification method (LVSV–PuSV) [27], used as internal reference in the present study. Moreover, the presence of aortic diastolic flow reversal in the descending aorta as well as left ventricular dilatation have been suggested to enhance the diagnostic certainty [44].

Our study has some limitations. Firstly, the study comprised only a relatively small number of patients, which has nonetheless been sufficient to show significantly differences concerning the impact of complex flow on the quantification of RVol and RF using PC-MRI. Secondly, the selected patient cohort did not include patients with mild AR. Future studies are warranted to include a larger range of AR severity. Thirdly, there is no “golden standard” to determine the true RVol and RF. In the present study, we chose the indirect method (LVSV–PuSV) as internal reference, accepting an offset bias. Finally, 4D flow measurements with retrospective gating may overcome some of the challenges in assessing AR severity in regions of complex flow, but this needs to be evaluated in future studies.

In conclusion, this study shows that quantification of AR using PC-MRI is influenced by complex flow, associated with dilatation of the aorta, which can lead to underestimation of the severity grade. This in turn can compromise the clinical decision making and the timing of surgery, and as such, alternative MRI methods e.g. LVSV–PuSV should be included in the examination protocol to ensure diagnostic certainty for patients with enlarged aortas.

## Data Availability

Data available on request from the authors.
